# Exploring the impact of social network change: Experiences of older adults ageing in place

**DOI:** 10.1111/hsc.12846

**Published:** 2019-09-06

**Authors:** Willeke H. Vos, Leonieke C. van Boekel, Meriam M. Janssen, Roger T. A. J. Leenders, Katrien G. Luijkx

**Affiliations:** ^1^ Department Tranzo Tilburg School of Social and Behavioral Sciences Tilburg University Tilburg The Netherlands; ^2^ Department Organization Studies Tilburg School of Social and Behavioral Sciences Tilburg University Tilburg The Netherlands; ^3^ Jheronimus Academy of Data Science 's-Hertogenbosch The Netherlands

**Keywords:** older adults; social network change; ageing in place; impact; experiences

## Abstract

Social networks are sources of support and contribute to the well‐being of older adults who are ageing in place. As social networks change, especially when accompanied by health decline, older adults’ sources of support change and their well‐being is challenged. Previous studies predominantly used quantitative measures to examine how older adults’ social networks change. Alternatively, this study explores the impact of changing social networks on older adults’ lives by examining their personal experiences. We held four focus groups, two with a total of 14 older adults who are ageing in place and receiving home care and two with a total of 20 home‐care nurses from different regions and organisations in the Netherlands. Subsequently, an expert team of home‐care professionals and managers discussed and verified the results. Procedures for grounded theory building were used for analysis. We revealed four themes of high‐impact experiences: (a) struggling with illness/death of the spouse; (b) working out a changing relationship with (grand)children; (c) regretting the loss of people they have known for so long and (d) feeling dependent and stressed when helpers enter the network. Also, network dynamics were found to follow three consecutive stages: (a) awareness of social network change; (b) surprise when social network change actually occurs and (c) acceptance and adjusting to new circumstances. Together, the four themes of experiences and three stages of network change form an integrative model of the role of social network dynamics for older adults’ lives when ageing in place.

**What is known about this topic**

- Social networks contribute to older adults’ well‐being and are sources of support during ageing in place.
- Changing social networks might compromise older adults’ ageing in place.
- The literature on older adults’ changing social networks leaves older adults’ experiences underexposed.

**What this paper adds**

- Four themes of high‐impact experiences of social network change, that are often experienced simultaneously or consecutively, make older adults feel they are drifting away from the life they used to live.
- Three consecutive stages of social network change indicate the negative impact of, but also the recovery from social network change.

## 1 INTRODUCTION

Older adults prefer to live independently and to stay in their own home if possible, also called ‘ageing in place’ (Wiles, Leibing, Guberman, Reeve, & Allen, 2012). Social networks contribute to ageing in place directly, as providers of social support (Burt, 1997; Kahn & Antonucci, 1981) and indirectly, functioning as a buffer for effects of stressful events (Krause, 1986) and more generally as enablers of health and well‐being (Berkman, Glass, Brissette, & Seeman, 2000; Cohen & Wills, 1985; Cornwell & Laumann, 2015; Freund & Baltes, 1998; Huber et al., 2016; Jowkar, Friborg, & Hjemdal, 2010; Kawachi, 2001; Seeman, Lusignolo, Albert, & Berkman, 2001; Uchino, Cacioppo, & Kiecolt‐Glaser, 1996). Literature on older adults’ social network change indicates that social networks of older adults remain stable (Bowling, Grundy, & Farquhar, 1995; Conway, Magai, Jones, Fiori, & Gillespie, 2013; van Tilburg, 1998) or decline (Fuller‐Iglesias, Webster, & Antonucci, 2015; Huxhold, Fiori, & Windsor, 2013; Reinhardt, Boerner, & Benn, 2003), but rarely grow. Other common findings include a loss of friends and a relatively increasing share of family members within networks (Bowling et al., 1995; Conway et al., 2013; Fuller‐Iglesias et al., 2015; Reinhardt et al., 2003) and the ending of relationships without frequent contact or with little support (‘peripheral relations’) (Kahn & Antonucci, 1981; Klein Ikkink & van Tilburg, 1999). These changes in social networks, some of which can be interpreted as life events, challenge ageing in place because they are negatively associated with older adults’ health and well‐being (Ellwardt, van Tilburg, Aartsen, Wittek, & Steverink, 2015; Ellwardt, van Tilburg, & Aartsen, 2015; Kelly et al., 2017; Krause, 2004) and they affect support needs and resources (Guiaux, van Tilburg, & Broese van Groenou, 2007; Tomassini et al., 2004). The current literature contains many studies of change in older adults’ social networks, but leaves older adults’ experiences underexposed. We believe that examining older adults’ experiences with their changing social networks from a more holistic and comprehensive approach, could contribute to a broader understanding of social network change in older adults’ lives.

European welfare states with ageing populations promote substitution from expensive forms of care, such as hospital or residential care, to less expensive forms, such as ageing in place with additional support (Pacolet, Bouten, & Versieck, 2018). In the Netherlands, this substitution appears to be successful because long‐term care health expenses are deflected, although it raises questions about the quality of life of older adults’ ageing in place (Kromhout, Kornalijnslijper, & Klerk, 2018). Home‐care nurses support older adults in continuing to age in place. In the Netherlands, home‐care nurses are therefore assigned to focus (a.o.) on the social networks of older adults (Blok & van Rijn, 2014; V&VN & B. V. V. N., 2^2012^). However, older adults are a heterogeneous group with a large diversity in their social networks and with continually changing care paths (Campen, Broese van Groenou, Deeg, & Iedema, 2013). This challenges home‐care nurses, and other professionals who enhance ageing in place, to give the right support at the right moment. A more comprehensive understanding of how older adults experience the impact of changes in their social networks on their lives could benefit home‐care nurses in the speed and accuracy of diagnoses (Gordon, 1994; Paans, Sermeus, Nieweg, Krijnen, & van der Schans, 2012) and in the customisation of their support.

Hence, this study aims to explore older adults’ experiences of changes in their social networks and to understand the impact of these changes on their lives.

## 2 METHODS

### 2.1 Design

We held two focus groups with older adults; one with ten participants in a rural area (a municipality with 36,000 inhabitants) and one with four participants in a city in the Netherlands (>220,000 inhabitants) to maximise input variation within a rather homogenous sample (Miles, Huberman, & Saldana, 2018). The older adults were selected through four home‐care organisations within the university region, with the inclusion criteria of being 65 years or older and ageing in place. They received information from their home‐care nurse about the study. After signing and returning the informed consent form, they were invited by the researchers.

We held two additional focus groups with home‐care nurses, to get additional detail on older adults’ perspectives on social network change (Creswell & Miller, 2000), both in the rural area and the city. Each had 10 participants, who were selected through the same four home‐care organisations, with the inclusion criterion of working predominantly with older adults. The nurses received information about the study through their manager. After signing and returning the informed consent form, they were invited by the researchers. Most home‐care nurses who participated also recruited older adults (Table 1).

Finally, after the focus groups, an expert group discussion was organised to check our results (Creswell & Miller, 2000; Miles et al., 2018) and enrich findings. The experts were recruited with the inclusion criterion of working directly or indirectly with older adults and were selected through convenience sampling within the network of the research group (Miles et al., 2018).

Ethics approval was given by the Ethics Review Board of the School of Social and Behavioral Sciences of Tilburg University (EC‐2017.15t).

**Table 1:** Focus group respondents

Older adults		Home‐care nurses	
*n* (%)		*n* (%)	
Sex	14	Sex	20
Women	8 (57%)	Women	19 (95%)
Men	6 (43%)	Man	1 (5%)
Age	10	Age	16
60–69	2 (20%)	20–29	3 (19%)
70–79	4 (40%)	30–39	2 (13%)
80–89	3 (30%)	40–49	3 (19%)
90–99	1 (10%)	50–59	6 (36%)
		60–69	2 (13%)
Marital status	14	#years in elder care	16
Married	9 (64%)	≥ 40 years	3 (19%)
Partner deceased	5 (36%)	≥30 to <40 years	4 (24%)
		≥20 to <30 years	3 (19%)
		≥10 to <20 years	3 (19%)
		<10 years	3 (19%)
(Grand)children	14	# years home‐care nurse	16
Yes	14 (100%)	≥40 years	2 (13%)
		≥30 years	3 (19%)
		≥20 to <30 years	3 (19%)
		≥10 to <20 years < 10 years	8 (49%)
# years home care	12	#contract hours a week	15
>5 years	6 (50%)	32–36 hr/week	8 (53%)
>1 to ≤5 years	3 (25%)	24–31 hr/week	6 (40%)
≤1 year	1 (8%)	<24 hr/week	1 (7%)
0 years	2 (17%)		
Educational level	12	Educational level	15
High (ISCED level ≥ 5)	3 (25%)	Applied science (ISCED levels 5–6)	14 (93%)
Middle (ISCED levels 3–4)	3 (25%)	Secondary vocational (ISCED level 4)	1 (7%)
Low (ISCED levels 0–2)	6 (50%)		
Notes: Participants all met inclusion criteria of age ≥ 65 and ageing in place, except one (age 64) The two respondents with 0 years of home care were volunteers at the focus group location. Due to a large no show they offered to participate.		Notes: Participants all met inclusion criteria: working as a home‐care nurse, predominantly with older adults Four participants eventually did not return their demographics form	

### 2.2 Data collection

In the focus groups, older adults were asked to reflect on how they experienced the changes in their social networks. Home‐care nurses were asked to reflect on the changing social networks they observed in their daily work with older adults. The experts were asked to compare the preliminary results of this study to observations and experiences from their own work environments.

An exploratory approach was used to guide the focus groups, to encourage participants to share their experiences with minimum guidance by the interviewers and with maximum space for self‐disclosure through discussion between participants (Freeman, 2006). The interview guide contained three topics. The first topic was ‘social networks’ and was used as a conversation starter and as a way to clarify and reach consensus on the social network concept. The second topic was ‘social network change’. Respondents were invited to share their experiences with social network change, sometimes stimulated by some in‐depth questions such as ‘I hear you have recently experienced several losses. Could you tell us more about that period?’ or ‘You told us things become harder. Can you explain what you mean by that’? The third topic was about how changing social networks impacted their lives. The focus groups were operated by two moderators (WV and LB) to ensure reliability (Morrison‐Beedy, Côté‐Arsenault, & Feinstein, 2001) and a research assistant took notes to support verbatim transcription. The focus group discussions took approximately two hours each. Three out of four focus groups were audio‐taped and transcribed verbatim. One focus group was reproduced by research notes due to failure of audio‐equipment. Focus groups were held in April and May 2017. The expert group discussion was held in November 2017 and lasted approximately 30 min, as part of a larger meeting. The meeting was audio‐taped and discussion themes were abstracted and transcribed non‐verbatim.

### 2.3 Data analysis

Data analysis followed procedures for grounded theory building (Corbin & Strauss, 2014) because of its usefulness for understanding specific phenomena (i.e. experiences of social network change) within processes (i.e. ageing), using eclectic data (i.e. individual level data, collected through focus groups) (Langley, 1999). First, two researchers (WV and LB) independently performed open coding of the transcripts of the focus groups with older adults and then discussed this until a preliminary set of codes remained. The preliminary set of codes was discussed by all the authors and further refined into a definitive set. Second, these codes were clustered, and conceptual themes were defined. As a third step, WV and LB evaluated the codes within the conceptual themes: codes were clustered and named when they described similar experiences (‘lower‐level concepts’). An example of a lower level concept within a conceptual theme is: ‘support by children increases’ within ‘a change in relations between people in the network’. The fourth step was to search for process indicators; we searched for text fragments in which older adults explicitly mentioned that ‘x caused y’ or that ‘y preceded z’. This step resulted in a small list of relations between lower‐level concepts. During data analysis, intermediate results and the next steps were continuously discussed by all authors jointly. As step five, the focus groups with home‐care nurses were coded and preliminary results were enriched. Finally, the expert group discussed preliminary results, based on their practice, and preliminary results were verified or falsified and enriched further.

## 3 RESULTS

Older adult participants were ageing in place with (in)formal support and were between 64 and 92 years old. Most participants had received home care for more than a year and had a network with children, grandchildren, neighbours and friends; one participant had no grandchildren.

The participating home‐care nurses were between 22 and 63 years old. The majority had a degree in applied nursing sciences, worked mainly with older adults and had over 5 years working experience in this profession.

Experts were a social worker (one), home‐care nurses (three), managers (three) and a public health advisor (one). No additional demographic details were collected. The experts used experiences from their own practices to discuss preliminary results.

### 3.1 Four high‐impact experiences of social network change

The experiences of older adults with social network change revealed four conceptual themes that impact older adults’ lives the most.

#### 3.1.1 Theme 1: Struggling with the illness or death of the spouse

Repeatedly, older adults emphasised the importance of the spouse in their lives and how illness or death impacts them. Older adults showed strong norms and beliefs about partnership and marriage, even if one's health is at stake. An older man (respondent 9, age 83), who takes care of his wife because she has dementia, shared with us:

Yes, it takes a lot of effort [WV: to take care of his wife], but I don't mind. I have chosen, I always say, to… [gets emotional] … to share joys and sorrows with my wife.

When the partner becomes ill, spouses experience social isolation and exhaustion as a result, and they neglect their own physical and emotional needs. It often takes a crisis with the ill partner to stop this lapse of caring and suffering. Both home‐care nurses and members of the expert group mentioned the strong unity of marital partners and the fragility of this unity when a partner becomes ill. Older adults expressed the importance of maintaining their individual activities (without a partner); this provides topics for daily conversations between the partners and a moment of relaxation. However, when one of the partners becomes ill, it takes effort to keep enjoying one's own activities—the ill partner must be taken care of, and chores must be finished. Nevertheless, participants agreed that the effort is worth making, like the older man (respondent 9, age 83) who takes care of his wife:

She [WV: his wife] goes to a daycare facility for three days a week. On Mondays, I go cycling. But I do have to arrange with my children: ‘are you home then?’. Because she is home at 4 p.m. and I'm not always back at that time. Yes, well, and that's how it goes.

When the spouse dies, first there is the grief of losing a life‐long partner. Then, a growing awareness rises about being alone. Finally, the loss becomes part of life but the pain or grief never goes away. One of the ‘younger’ older women (respondent 2, age 70) told us about her experiences after her husband died:

In September, it will be seven years [WV: since her husband died]. And it is still hard. But, ehm, hard without sharp edges. This is a way of comforting you [WV: nodding to Mrs. M]. Without the hard sides, without thorns. The loss gets worse. But it doesn't sting anymore, like before. These sharp edges…, and now you can talk about the fun things, but also about the less fun things. But it never gets…. No, it never goes away. It does not become any better.

Home‐care nurses point out that there are differences between men and women experiencing spousal loss, with women needing less time to recover from their spouse's death than men. Home‐care nurses observe that women continue to spend time with friends after the loss of their partner, whereas men tend to spend more time with their (grand)children.

The older adults experienced their struggling with the illness or death of their spouse as the most impactful social network change in their lives. They also note that this does not happen alone and is often accompanied by additional types of experiences that we describe in the next sections.

#### 3.1.2 Theme 2: Regretting the loss of people they have known for so long, despite finding new contacts

Older adults often indicated they were losing friends and family members of their own age with whom they had had long‐lasting and often frequent contact. These people are lost because they die, but also because they do not come to visit them anymore because they were uncomfortable with illness or because they had become ill themselves. Maintaining existing friendships appears to be challenging, because of the decreased mobility and dependency on carers. Network decline tended to be lower if the remaining spouse was the one responsible for maintaining friendships during their marriage. Age also tended to be a factor of importance in experiencing network decline. The youngest participant (respondent 13, age 64), reported that he has lots of friends and an older lady (respondent 11, age 86) replies:

That is because this man is younger, I mean you are a generation younger than we are, so that is why he has more contacts. We used to have that too! Can I say that one generation makes a difference?

Older adults also referred to new friends coming into their networks, for example by joining a club or community. They value these positive experiences highly. However, overall, their losses dominated the focus group conversations, leaving them with feelings of melancholy and sadness. As one of the younger male respondents (respondent 1, age 73) sighed:

And, well, then I became ill, and then things changed because I stopped with this voluntary union committee. Yes, I gave it up. I was forced to make this decision. That I really regretted.

Home‐care nurses observed barriers and facilitators for establishing new relations. Barriers are physical impairment (mobility, reduced hearing capacity), limited flexibility in give‐and‐take (drinking coffee with others is nice, but only at 10 a.m.) and rather closed communities (not being one of them). Facilitators are a location nearby to meet others and the help of someone who connects people and helps older adults to overcome their barriers. Members of the expert group noticed that adults often end up relatively far from ‘home’ in a rather homogeneous group of older adults when children insist on relocation for safety reasons. This situation limits older adults in maintaining contact with those they have known for so long and incapacitates them in making contact with new neighbours because these face similar barriers to establishing new relations.

#### 3.1.3 Theme 3: Working out a changing relationship with children and grandchildren

Older adults were reluctant to ask their children for help, but sometimes circumstances force them. Older adults should not rely on their children was a shared belief, because the children have their own lives. Nevertheless, asking help from their children appears inevitable for older adults and as parents they have to accept this. Common forms of support by children are advice and practical support, such as taking care of financial issues, buying clothes, looking after the ill parent, and teaching them computer skills. One older woman (respondent 2, age 70) was very firm in her views on this subject:

When my husband died I said one thing to my children ‘I will never lean on you’. Sometimes it is very hard when I sit there, and I think ‘Yes, well, of course, I can ask my daughter and my son‐in‐law and my grandchildren.’ But everyone is busy, busy, busy, nowadays.

Home‐care nurses observed differences between families with higher and lower socioeconomic status (SES) in how children support their parents. In high‐SES families, older adults and their children frequently rely on professionals for support, whereas low‐SES families seem to have more people in their neighbourhood who support them. Members of the expert group noted that higher SES and city families seemed to be more familiar with organising and buying help at a distance, while lower SES and rural families seemed to be more often present to provide help themselves.

Sunday afternoon visits by (grand)children are cherished moments, but older adults also feel they cannot keep up anymore. They find it hard to follow and participate in conversations and feel that family members' attention to them is always divided due to ‘that thing’ (smartphone). Respondents 9, 8 and 2 agreed upon this respectively:

There's no fun anymore, when the children come home, there's no fun. (9).

No, not with such a thing [WV: a smartphone] stuck under their noses! (8).

(…) [WV: when her grandson visits her] He is barely inside my house and then he grabs that thing. Then I say: “what do we do? Put that thing off, if only for 15 min!” Away with it! (2).

The continuous digital availability feels like a loss of freedom. However, contact via e‐mail and WhatsApp makes participants feel less lonely when by themselves because (grand)children are only one push‐on‐a‐button away. A quote from one of the older males (respondent 6, age 92) illustrates this very well:

Then we [WV: he and his son] decided to, how do you call it, learn the computer. On a Sunday afternoon, my son came and visited me, and he brought a computer. ‘Well, just start up, Dad!’ he said. […] And I said, ‘You've convinced me!’. And I did [WV: learn computer skills] because my oldest son moved to New Zealand 25 years ago. Now we can Skype with each other.

Home‐care nurses observed that the use of electronic applications in caring, such as an electronic patient file, is beneficial for involving family members in the caring network. They also observed an increasing presence of grandchildren in the network, due to the digitalisation of family contacts.

Although reversing relationships and generational differences between them and their children take older adults out of their comfort zone, they also breed feelings of thankfulness and of being part of present times. That can be challenging, but also satisfying.

#### 3.1.4 Theme 4: Feeling dependent and stressed when helpers enter the social network

Helpers are members of the network who provide support to older adults, such as taxi driving. Older adults did not differentiate between formal and informal support or between paid and unpaid helpers. Helpers can be new to the network (such as home‐care nurses) and new relations can be formed with people who were already present in the network (such as a child or neighbour who starts doing the grocery shopping). Older adults feel dependent when helpers enter the network. It causes stress. Older adults try to reduce dependency by maintaining reciprocity, for example by compensating helpers' fuel costs, returning favours, and expressing thankfulness. Home‐care nurses were not very aware of the dependency experienced by older adults as a result of their caring. A striking example of dependency and stress is given by a lady (respondent 8, age is missing) who is taking care of her husband who wears a catheter.

The point is, we agreed, when someone with Alzheimer's disease has to go [WV: to the toilet], he has to go. He will start to sprinkle all over the place because he will go to the toilet himself! So, we agreed on 9 and 10 a.m. [WV: with the home care nurse] Sometimes they won't make it by then. And he does not understand that. I tell him: you can't do it yourself. He orders me to help him myself. I can do that, but if I cause an infection… And I want to protect him from that.

A special type of helper appeared to be the helper with whom one can discuss important issues about the difficulties of being an informal carer or about the spouse's illness and its broader consequences. This helper is considered a trusted member of the caring network and older adults referred to this kind of helper as ‘very important’ and ‘necessary to continue the status quo’. The relationship with this helper strengthens over time through frequent contact. Home‐care nurses mentioned that older adults often share feelings and emotions with them. Expert group members acknowledged that merely being present in the older adults' networks, listening to them and asking questions about their lives, can help older adults establish new contacts themselves and feel a little better about life.

For older adults, the devil appears to be in the combination. They struggle with the loss of self‐chosen contacts with whom they share a history and have to work out new (somehow imposed) contacts and relations. This tilting ratio in their networks makes older adults feel they are drifting away from the life they used to live.

### 3.2 Three stages of social network change

Even though the older adults each have their distinctive lives, the experiences that were discussed in the focus groups appear to follow a similar process. In the first stage, before social network change, findings indicate that older adults are aware of the changing nature of their social networks, perceiving it as part of growing old. Older adults reported that they prepared themselves for their ‘old age’ through activities such as signing up for smaller housing, making agreements with the children about future support or by focusing on neighbourhood activities. As a widowed older woman (respondent 12, age is missing) told us:

…but we arranged that before my husband died. My husband wasn't ill, but he was 88, so, I only want to say that we had to take care of things. So we organized sort of a party, with cake, a nice little gathering. And then we agreed on one son doing the financial stuff, another one doing the taxes and my daughter would help me with my women's stuff (buying clothes, grocery shopping). Well, we made arrangements in August and in the first week of January he died.

The extent to which older adults (can) prepare themselves for their old age seems related to the size and composition of their social networks in earlier life and their health status. Focus group participants expressed the need to have and maintain a mix of contacts that are meaningful and reciprocal with contacts that are superficial and casual. Contacts are not only used for a nice conversation but also as a distraction from their situation and to have something to look forward to. As a widowed woman (respondent 2, age 70) confessed with a smile:

Then [WV: when in need of contacts] I go and have fun at the knitting café. To have a nice chat. I have to tell you: you can't bring any difficult knitting, because … well [WV: it's hard to pay attention to the knitting, while chatting all the time!].

In the second stage, during social network change, even if they had prepared for pending changes, older adults are still caught off‐guard when their social networks started changing. These network changes are often accompanied (or preceded) by an acute health change and they feel as if their life is turned upside‐down. In retrospect, older adults concluded that the changes in their social networks greatly impacted their lives. Home‐care nurses brought up crisis situations that arise when one of the partners becomes ill or when incidents happen due to accumulated health decline—not only hospitalisation or death occurring, but also full support or even institutionalisation had to be arranged for the partner that remained. One woman (respondent 7, age is missing) told us quite emotionally about the very recent admission of her husband to a nursing home, after a long period of incremental health decline in which she provided informal care for her husband:

No. I don't want this either. But I can't take it any longer! I can't take it anymore. I have no rest anymore; I can't sleep anymore!

And one of the male respondents (respondent 1, age 73) in reviewing his recent past*: Before my, let*'*s say ‘episode’ (WV: of illness), in those days I went out with friends*.* I went away for the weekend, for example, or visited some place*.* But those things, I do miss that*.* Look, those contacts are gone, of course*.* Not entirely gone, but we don*'*t meet on a regular basis anymore*.

In the third and final stage, after social network change, older adults feel that they are ‘moving to the sidelines’ in life, although they generally feel well. Participants revealed modest expressions of satisfaction about their general living conditions (housing, care, support) and their contacts. This satisfaction appears to be a form of resignation which is prompted by a feeling of being unable to change the situation and the inevitability of ‘letting things go’. Participants indicated that acceptance of the situation facilitated them in becoming fairly satisfied again, which they illustrated with examples of going out again, finding new network contacts, and participating in new activities. Successful adjustment after network change appears to contribute to becoming satisfied again. Still, accepting the changes and adjusting to its consequences is experienced as tough by older adult participants. As one of the older women reported (respondent 8, age is missing):

And when you fall ill, my husband has Alzheimer's disease, then you lose a lot of people, who don't want to come anymore. Yes, well, I'm fine with that. Of course, I'm not fine with that, but this is how it is, you can't do anything about that.

And one of the male respondents (respondent 9, age 83) said: *As long as you feel healthy and well*.* That*'*s what it is all about; it*'*s not about the difficulties*.

### 3.3 A model of the impact of social network change

**Figure 1:**
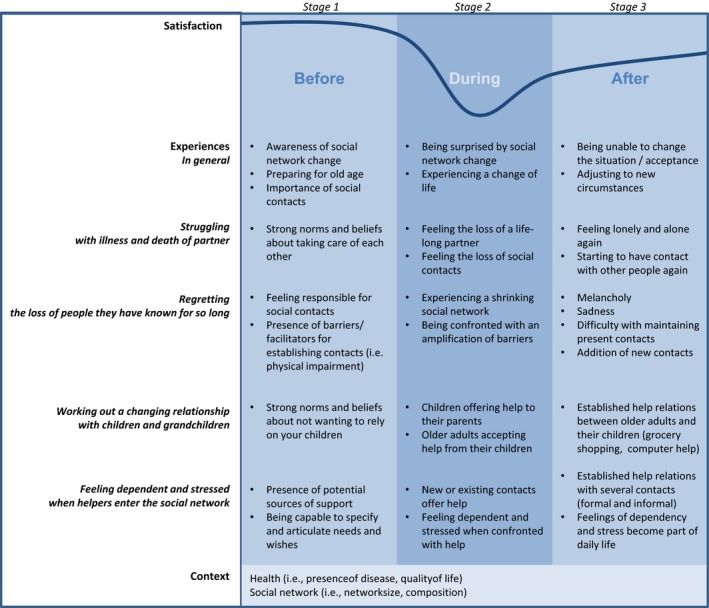
An integrative model of the impact of social network change on older adults’ lives

Experiences of social network change appear to follow a comparable, general process comprising three stages (described above). In order to reach a more comprehensive understanding of the impact of social network change on older adults' lives, in Figure 1 we show how the three stages interact with the four themes of high‐impact experiences (‘struggling with death/illness’, ‘regretting loss’, ‘working out changing relations’, and ‘feeling dependent’). Although the occurrence of the experiences and the order of the stages appear to apply quite generally in our sample, during focus group conversations we noticed large individual differences in intensity and pace of experiences between older adults. Also, we noticed large differences in the size and composition of their networks and in their health statuses.

Figure 1 shows how social network change impacts older adults' lives. The four themes of experiences of social network change vary in relative intensity. Moreover, when multiple experiences appear simultaneously in older adults' lives, it makes them feel they are drifting away from the life they used to live. The consecutive stages of social network change indicate that life can be tough for older adults when experiencing social network change, but that they do tend to find ways to recover to a certain extent. The model suggests that life satisfaction moves along this line in a predictable swing. In the end, it is in the pace and intensity of this process where the (main) individual differences between older adults occur.

## 4 DISCUSSION

This study aimed to explore how older adults experience the changing of their social networks and how these experiences impact their lives—as shared by themselves and home‐care nurses. The findings reveal four themes of experiences regarding social network change that impact strongly on the older adults' lives. We also find that these experiences follow three consecutive stages. Below, we discuss the contribution to the field these results make and how home‐care nurses can benefit from the results to customise their support of older adults.

The four high‐impact social network changes that were found in our study corroborate findings of other studies on the impact of the loss of loved ones (Carr, House, Wortman, Nesse, & Kessler, 2001; Donnelly & Hinterlong, 2010; Fry, 1998; Van Baarsen, Van Duijn, Smit, Snijders, & Knipscheer, 2002; Van Grootheest, Beekman, Broese van Groenou, & Deeg, 1999) and on developing relations between older adults and their informal and formal carers (Byrne, Goeree, Hiedemann, & Stern, 2009; Egdell, 2012; Wellman, Wong, Tindall, & Nazer, 1997). Also, we found experiences and perceptions that are in line with other studies, such as the loyalty of spouses, acceptance of oneself and the situation, feelings of loneliness (Reichstadt, Sengupta, Depp, Palinkas, & Jeste, 2010; Roelofs, Luijkx, & Embregts, 2017), and the importance of connectedness, participation and independence (Bruggencate, Luijkx, & Sturm, 2018). In addition, our study extends findings in the field, first, by reaching a more comprehensive understanding of the complexity of older adults' experiences. Struggling with loss (of self‐chosen, life‐long contacts with considerable depth) and being compelled to work out new contacts (which are somehow imposed and often with less depth) makes older adults feel they are drifting away from the life they used to live. Also, our study provides insight into the heterogeneity of experiences of older adults' ageing in place, despite having rather homogeneous focus groups of older adults. Adding to the earlier finding in the field that gender and SES are responsible for a variety of network outcomes (Broese van Groenou, Glaser, Tomassini, & Jacobs, 2006; Broese van Groenou & van Tilburg, 2^2003^; van Grootheest et al., 1999) we found indications that gender and SES may fuel a variety in experiences of change. ‘For example we found that older adults with a low SES appear to have more people in their neighbourhood to support them than older adults with a high SES, which corroborates findings from a study across four European countries in which older adults with a low SES receive more informal help than older adults with a high SES (Broese van Groenou, Glaser, Tomassini, & Jacobs, 2006). However, according to de Informal Care Model (ICM), it's not just the onset of informal care provision that varies with SES, but also the need for care, care giver's dispositions, older adults' social networks and the presence of—or cutbacks on—community care facilities (Broese van Groenou & De Boer, 2016). We perceive that the factors from the ICM are not only responsible for the onset of informal care provision, but also for a large diversity in older adults' experiences of change while ageing in place. In this study we incorporated perspectives of both older adults and home‐care nurses and considered this to be of added value since older adults' experiences are necessary for understanding the—differences in—impact of social network change, while home‐care nurses perspectives are needed to consider individual experiences in a broader context. However, in order to really benefit home‐care nurses, further research is necessary to find the origins of these differences in experiences and break down a large, and growing, population into subgroups that are suitable for customised intervention.

The three consecutive stages of social network change that emerged in our study relate to well‐known ageing theories, such as social convoy theory (Kahn & Antonucci, 1981), socioemotional selectivity theory (Carstensen, 1992), and selective optimisation with compensation (Baltes & Baltes, 1990). For example our study found indications that older adults' are aware of the changes in their social networks that might occur in the future and that they do undertake activities to prepare themselves for the inevitable changes. This aligns with findings that older adults are aware of age‐related gains and losses and of the limited time they have left in this world and that they are capable of setting goals in life and adapt their behaviour with these goals in mind (Baltes & Baltes, 1990; Löckenhoff & Carstensen, 2004). However, ageing theories state that older adults have age‐related advantages that enable them to regulate their emotions and that these advantage are at stake in periods of (unavoidable) distress (Blanchard‐Fields, 2007; Charles & Piazza, 2009). Our study contributes to ageing literature by indicating that unavoidable periods of distress (when changes in social networks occur) might impact older adults' lives negatively, but that they can recover and find ways to continue successful ageing (albeit at lower satisfaction levels than before—see Figure 1). Home‐care nurses are often brought in to provide support in these periods of distress. Having knowledge about the consecutive stages of social network change could prevent them from hastening into profound and perhaps sometimes unnecessary decisions, such as giving up ageing in place. However, in learning more about how ageing in place works for older adults, we might need to reconsider ageing in place as an ultimate goal. Further research on what works for older adults, in what circumstances and how (Pawson & Tilley, 1997) could inform policy and practice to be better able to personalise goals concerning ageing in place.

## 5 STRENGTHS AND LIMITATIONS

A strength of this study is that it provides valuable insights into those at stake from multiple perspectives. Another strength of our qualitative approach is that it gives additional meaning to the quantitative studies in the literature. Limitations of this study are the limited sample size and the overrepresentation in our sample of married and widowed older adults with (grand)children. Also, discussing the impact of social network change in a group‐setting might have burdened older adults to express their most profound emotions. Despite that, the findings appear to be robust, because the themes in older adults' focus groups were also central in the focus groups with home‐care nurses and, additionally, were confirmed by the expert group members and in the literature.

## 6 CONCLUSION

This study focused on exploring older adults' experiences of social network change and the impact of these experiences on their lives. Results indicate four themes of high‐impact experiences and three stages of social network change. The personal impact of these experiences is determined by the intensity and pace of the experiences and by older adults' health and social networks at the outset of change. Findings complement existing literature with a broader understanding of the impact of social network change on older adults' lives and can benefit home‐care nurses in customisation of formal and informal support.

## References

[hsc12846-bib-0001] Baltes, P. B. , & Baltes, M. M. (1990). Psychological perspectives on successful aging: The model of selective optimization with compensation. Successful Aging: Perspectives from the Behavioral Sciences, 1(1), 1–34. 10.1017/CBO9780511665684.003

[hsc12846-bib-0002] Berkman, L. F. , Glass, T. , Brissette, I. , & Seeman, T. E. (2000). From social integration to health: Durkheim in the new millennium. Social Science & Medicine, 51(6), 843–857. 10.1016/S0277-9536(00)00065-4 10972429

[hsc12846-bib-0003] Blanchard‐Fields, F. (2007). Everyday problem solving and emotion: An adult developmental perspective. Current Directions in Psychological Science, 16(1), 26–31. 10.1111/j.1467-8721.2007.00469.x

[hsc12846-bib-0004] Blok, S. A. , & van Rijn, M. J. (2014). Kamerbrief over de transitieagenda langer zelfstandig wonen (2014–0000299501), Ministry of the Interior and Kingdom Relations (webversion). Retrieved from https://www.rijksoverheid.nl/documenten/kamerstukken/2014/06/04/kamerbrief-over-langer-zelfstandig-wonen

[hsc12846-bib-0005] Bowling, A. , Grundy, E. , & Farquhar, M. (1995). Changes in network composition among the very old living in inner London. Journal of Cross‐Cultural Gerontology, 10(4), 331–347. 10.1007/BF00972333 24389882

[hsc12846-bib-0006] Broese van Groenou, M. I. , & De Boer, A. (2016). Providing informal care in a changing society. European Journal of Ageing, 13(3), 271–279. 10.1007/s10433-016-0370-7 27610055 PMC4992501

[hsc12846-bib-0007] Broese van Groenou, M. , Glaser, K. , Tomassini, C. , & Jacobs, T. (2006). Socio‐economic status differences in older people's use of informal and formal help: A comparison of four European countries. Ageing & Society, 26(5), 745–766. 10.1017/S0144686X06005241

[hsc12846-bib-0008] Broese van Groenou, M. , Glaser, K. , Tomassini, C. , & Jacobs, T. (2006). Socio‐economic status differences in older people's use of informal and formal help: A comparison of four European countries. Ageing and Society, 26(5), 745–766. 10.1017/S0144686X06005241

[hsc12846-bib-0009] Broese van Groenou, M. , & van Tilburg, T. (2003). Network size and support in old age: Differentials by socio‐economic status in childhood and adulthood. Ageing & Society, 23(5), 625–645. 10.1017/S0144686X0300134X

[hsc12846-bib-0052] Bruggencate, T. , Luijkx, K. , & Sturm, J. (2018). Social needs of older people: A systematic literature review. Ageing and Society, 38(9), 1745–1770. 10.1017/S0144686X17000150

[hsc12846-bib-0010] Burt, R. S. (1997). A note on social capital and network content. Social Networks, 19(4), 355–373. 10.1016/S0378-8733(97)00003-8

[hsc12846-bib-0011] Byrne, D. , Goeree, M. S. , Hiedemann, B. , & Stern, S. (2009). Formal home care, informal care, and family decision making. International Economic Review, 50(4), 1205–1242. 10.1111/j.1468-2354.2009.00566.x

[hsc12846-bib-0012] Campen, C. , Broese van Groenou, M. I. , Deeg, D. J. H. , & Iedema, J. (2013). Met zorg ouder worden (Webversion). Retrieved from https://www.scp.nl/Publicaties/Alle_publicaties/Publicaties_2013/Met_zorg_ouder_worden

[hsc12846-bib-0013] Carr, D. , House, J. S. , Wortman, C. , Nesse, R. , & Kessler, R. C. (2001). Psychological adjustment to sudden and anticipated spousal loss among older widowed persons. The Journals of Gerontology: Series B, 56(4), S237–S248. 10.1093/geronb/56.4.S237 11445616

[hsc12846-bib-0014] Carstensen, L. L. (1992). Social and emotional patterns in adulthood: Support for socioemotional selectivity theory. Psychology and Aging, 7(3), 331–338. 10.1037/0882-7974.7.3.331 1388852

[hsc12846-bib-0015] Charles, S. T. , & Piazza, J. R. (2009). Age differences in affective well‐being: Context matters. Social and Personality Psychology Compass, 3(5), 711–724. 10.1111/j.1751-9004.2009.00202.x

[hsc12846-bib-0016] Cohen, S. , & Wills, T. A. (1985). Stress, social support, and the buffering hypothesis. Psychological Bulletin, 98(2), 310–357. 10.1037/0033-2909.98.2.310 3901065

[hsc12846-bib-0017] Conway, F. , Magai, C. , Jones, S. , Fiori, K. , & Gillespie, M. (2013). A six‐year follow‐up study of social network changes among African‐American, Caribbean, and U.S.‐born Caucasian urban older adults. International Journal of Aging and Human Development, 76(1), 1–27. 10.2190/AG.76.1.a 23540157

[hsc12846-bib-0018] Corbin, J. , & Strauss, A. L. (2014). Basics of qualitative research (4th edn). Thousand Oaks, CA: Sage Publications.

[hsc12846-bib-0019] Cornwell, B. , & Laumann, E. O. (2015). The health benefits of network growth: New evidence from a national survey of older adults. Social Science & Medicine, 125, 94–106. 10.1016/j.socscimed.2013.09.011 24128674 PMC3975821

[hsc12846-bib-0020] Creswell, J. W. , & Miller, D. L. (2000). Determining validity in qualitative inquiry. Theory into Practice, 39(3), 124–130. 10.1207/s15430421tip3903_2

[hsc12846-bib-0021] Donnelly, E. A. , & Hinterlong, J. E. (2010). Changes in social participation and volunteer activity among recently widowed older adults. Gerontologist, 50(2), 158–169. 10.1093/geront/gnp103 19556394

[hsc12846-bib-0022] Egdell, V. (2012). Development of support networks in informal dementia care: Guided, organic, and chance routes through support. Canadian Journal on Aging, 31(4), 445–455. 10.1017/S0714980812000323 23021103

[hsc12846-bib-0023] Ellwardt, L. , Van Tilburg, T. G. , & Aartsen, M. J. (2015). The mix matters: Complex personal networks relate to higher cognitive functioning in old age. Social Science & Medicine, 125, 107–115. 10.1016/j.socscimed.2014.05.007 24840784

[hsc12846-bib-0024] Ellwardt, L. , van Tilburg, T. , Aartsen, M. , Wittek, R. , & Steverink, N. (2015). Personal networks and mortality risk in older adults: A twenty‐year longitudinal study. PLoS ONE, 10(3), e0116731 10.1371/journal.pone.0116731 25734570 PMC4348168

[hsc12846-bib-0025] Freeman, T. (2006). ‘Best practice’ in focus group research: Making sense of different views. Journal of Advanced Nursing, 56(5), 491–497. 10.1111/j.1365-2648.2006.04043.x 17078825

[hsc12846-bib-0026] Freund, A. M. , & Baltes, P. B. (1998). Selection, optimization, and compensation as strategies of life management: Correlations with subjective indicators of successful aging. Psychology and Aging, 13(4), 531–543. 10.1037/0882-7974.13.4.531 9883454

[hsc12846-bib-0027] Fry, P. S. (1998). Spousal loss in late life: A 1‐year follow‐up of perceived changes in life meaning and psychosocial functioning following bereavement. Journal of Personal and Interpersonal Loss, 3(4), 369–391. 10.1080/10811449808409711

[hsc12846-bib-0028] Fuller‐Iglesias, H. R. , Webster, N. J. , & Antonucci, T. C. (2015). The complex nature of family support across the life span: Implications for psychological well‐being. Developmental Psychology, 51(3), 277–288. 10.1037/a0038665 25602936 PMC4497824

[hsc12846-bib-0029] Gordon, M. (1994). Nursing diagnosis: Process and application. St Louis, MO: Mosby Inc.

[hsc12846-bib-0030] Guiaux, M. , Van Tilburg, T. , & Broese van Groenou, M. (2007). Changes in contact and support exchange in personal networks after widowhood. Personal Relationships, 14(3), 457–473. 10.1111/j.1475-6811.2007.00165.x

[hsc12846-bib-0031] Huber, M. , van Vliet, M. , Giezenberg, M. , Winkens, B. , Heerkens, Y. , Dagnelie, P. , & Knottnerus, J. (2016). Towards a ‘patient‐centred’ operationalisation of the new dynamic concept of health: A mixed methods study. British Medical Journal Open, 6(1), e010091 10.1136/bmjopen-2015-010091 PMC4716212 26758267

[hsc12846-bib-0032] Huxhold, O. , Fiori, K. L. , & Windsor, T. D. (2013). The dynamic interplay of social network characteristics, subjective well‐being, and health: The costs and benefits of socio‐emotional selectivity. Psychology and Aging, 28(1), 3–16. 10.1037/a0030170 23066804

[hsc12846-bib-0033] Jowkar, B. , Friborg, O. , & Hjemdal, O. (2010). Cross‐cultural validation of the Resilience Scale for Adults (RSA) in Iran. Scandinavian Journal of Psychology, 51(5), 418–425. 10.1111/j.1467-9450.2009.00794.x 20149146

[hsc12846-bib-0034] Kahn, R. L. , & Antonucci, T. C. (1981). Convoys of social support: a life course approach In Kiesler S. B., Morgan J. N. & Oppenheimer V. K. (Eds.), Aging: Social change (pp. 383–405). New York, NY: Academic Press.

[hsc12846-bib-0035] Kawachi, I. (2001). Social capital for health and human development. Development, 44(1), 31–35. 10.1057/palgrave.development.1110211

[hsc12846-bib-0036] Kelly, M. E. , Duff, H. , Kelly, S. , Power, J. E. M. , Brennan, S. , Lawlor, B. A. , & Loughrey, D. G. (2017). The impact of social activities, social networks, social support and social relationships on the cognitive functioning of healthy older adults: A systematic review. Systematic Reviews, 6(1), 259. 29258596 10.1186/s13643-017-0632-2 PMC5735742

[hsc12846-bib-0037] Klein Ikkink, K. , & van Tilburg, T. (1999). Broken ties: Reciprocity and other factors affecting the termination of older adults' relationships. Social Networks, 21(2), 131–146. 10.1016/S0378-8733(99)00005-2

[hsc12846-bib-0038] Krause, N. (1986). Social support, stress, and well‐being among older adults1. The Journal of Gerontology, 41(4), 512–519. 10.1093/geronj/41.4.512 3722737

[hsc12846-bib-0039] Krause, N. (2004). Lifetime trauma, emotional support, and life satisfaction among older adults. Gerontologist, 44(5), 615–623. 10.1093/geront/44.5.615 15498837

[hsc12846-bib-0040] Kromhout, M. , Kornalijnslijper, N. , & Klerk, M. D. (2018). Veranderde zorg en ondersteuning voor mensen met een beperking. Landelijke evaluatie van de Hervorming Langdurige Zorg (Webversion). Retrieved from https://www.scp.nl/Publicaties/Alle_publicaties/Publicaties_2018/Veranderde_zorg_en_ondersteuning_voor_mensen_met_een_beperking

[hsc12846-bib-0041] Langley, A. (1999). Strategies for theorizing from process data. Academy of Management Review, 24(4), 691–710. 10.5465/amr.1999.2553248

[hsc12846-bib-0042] Löckenhoff, C. E. , & Carstensen, L. L. (2004). Socioemotional selectivity theory, aging, and health: The increasingly delicate balance between regulating emotions and making tough choices. Journal of Personality, 72(6), 1395–1424. 10.1111/j.1467-6494.2004.00301.x 15509287

[hsc12846-bib-0043] Miles, M. B. , Huberman, A. M. , & Saldana, J. (2018). Qualitative data analysis: A methods sourcebook. Sage Publications: Thousand Oakes, CA.

[hsc12846-bib-0044] Morrison‐Beedy, D. , Côté‐Arsenault, D. , & Feinstein, N. F. (2001). Maximizing results with focus groups: Moderator and analysis issues. Applied Nursing Research, 14(1), 48–53. 10.1053/apnr.2001.21081 11172230

[hsc12846-bib-0045] Paans, W. , Sermeus, W. , Nieweg, R. M. , Krijnen, W. P. , & van der Schans, C. P. (2012). Do knowledge, knowledge sources and reasoning skills affect the accuracy of nursing diagnoses? a randomised study. BMC Nursing, 11(1), 11 10.1186/1472-6955-11-11 22852577 PMC3447681

[hsc12846-bib-0046] Pacolet, J. , Bouten, R. , & Versieck, K. (2018). Social protection for dependency in old age: A study of the fifteen EU member states and. Norway: Routledge.

[hsc12846-bib-0047] Pawson, R. , & Tilley, N. (1997). Realistic evaluation. London: Sage.

[hsc12846-bib-0048] Reichstadt, J. , Sengupta, G. , Depp, C. A. , Palinkas, L. A. , & Jeste, D. V. (2010). Older adults' perspectives on successful aging: Qualitative interviews. The American Journal of Geriatric Psychiatry, 18(7), 567–575. 10.1097/JGP.0b013e3181e040bb 20593536 PMC3593659

[hsc12846-bib-0049] Reinhardt, J. P. , Boerner, K. , & Benn, D. (2003). Predicting individual change in support over time among chronically impaired older adults. Psychology and Aging, 18(4), 770–779. 10.1037/0882-7974.18.4.770 14692863

[hsc12846-bib-0050] Roelofs, T. S. , Luijkx, K. G. , & Embregts, P. J. (2017). Love, intimacy and sexuality in residential dementia care: A spousal perspective. Dementia, 18(3), 936–950. 10.1177/1471301217697467 29149794

[hsc12846-bib-0051] Seeman, T. E. , Lusignolo, T. M. , Albert, M. , & Berkman, L. (2001). Social relationships, social support, and patterns of cognitive aging in healthy, high‐functioning older adults: MacArthur studies of successful aging. Health Psychology, 20(4), 243 10.1037/0278-6133.20.4.243 11515736

[hsc12846-bib-0053] Tomassini, C. , Kalogirou, S. , Grundy, E. , Fokkema, T. , Martikainen, P. , Broese van Groenou, M. , & Karisto, A. (2004). Contacts between elderly parents and their children in four European countries: Current patterns and future prospects. European Journal of Ageing, 1(1), 54–63. 10.1007/s10433-004-0003-4 28794702 PMC5502680

[hsc12846-bib-0054] Uchino, B. N. , Cacioppo, J. T. , & Kiecolt‐Glaser, J. K. (1996). The relationship between social support and physiological processes: A review with emphasis on underlying mechanisms and implications for health. Psychological Bulletin, 119(3), 488 10.1037/0033-2909.119.3.488 8668748

[hsc12846-bib-0055] V&VN (Dutch Nurses' Association) (2012). Expertisegebied Wijkverpleegkundige. Retrieved from https://www.venvn.nl/Portals/1/Nieuws/2013%20Documenten/20121106%20Expertisegebied%20wijkverpl.pdf

[hsc12846-bib-0056] Van Baarsen, B. , Van Duijn, M. A. , Smit, J. H. , Snijders, T. A. , & Knipscheer, K. P. (2002). Patterns of adjustment to partner loss in old age: The widowhood adaptation longitudinal study. Omega‐Journal of Death and Dying, 44(1), 5–36. 10.2190/PDUX-BE94-M4EL-0PDK

[hsc12846-bib-0057] Van Grootheest, D. S. , Beekman, A. T. , Broese van Groenou, M. , & Deeg, D. J. (1999). Sex differences in depression after widowhood. Do men suffer more? Social Psychiatry and Psychiatric Epidemiology, 34(7), 391–398. 10.1007/s001270050160 10477960

[hsc12846-bib-0058] van Tilburg, T. (1998). Losing and gaining in old age: changes in personal network size and social support in a four‐year longitudinal study. The Journals of Gerontology Series B: Psychological Sciences and Social Sciences, 53B(6), S313–S323. 10.1093/geronb/53B.6.S313 9826973

[hsc12846-bib-0059] Wellman, B. , Wong, R.‐Y.‐L. , Tindall, D. , & Nazer, N. (1997). A decade of network change: Turnover, persistence and stability in personal communities. Social Networks, 19(1), 27–50. 10.1016/S0378-8733(96)00289-4

[hsc12846-bib-0060] Wiles, J. L. , Leibing, A. , Guberman, N. , Reeve, J. , & Allen, R. E. (2012). The meaning of “aging in place” to older people. Gerontologist, 52(3), 357–366. 10.1093/geront/gnr098 21983126

